# Supplementary material for: Organizational barriers in HPV vaccination uptake: A cross-sectional study among health sciences students
Source: PLoS One. 2025 Jun 24;20(6):e0326694. doi: 10.1371/journal.pone.0326694 (PMC12186899; doi:10.1371/journal.pone.0326694)
Supplement: S1 Table — (DOCX) [file pone.0326694.s001.docx]

|  | | **Odds Ratio** | | **Standard error** | **95% Conf. Interval** | | **p- Value** |
| --- | --- | --- | --- | --- | --- | --- | --- |
| **Model I - Main Analysis** | | | | | | | |
| **Sex** | Male (ref) | | | | | | |
|  | Female | | 7,952 | 2.547 | 4,244 | 14,899 | 0.000 |
| **Age** |  | | 0.814 | 0,046 | 0.728 | 0.910 | 0.000 |
| **Smoking habit** | Non-smoker (ref) | | |  |  |  |  |
|  | Smoker | | 0.783 | 0.234 | 0.436 | 1,407 | 0.415 |
|  | Former Smoker | | 0.559 | 0.325 | 0.179 | 1,748 | 0.318 |
| **Year of enrolment** | 1st (ref) | | | | | | |
|  | 2nd | | 0.893 | 0.348 | 0.416 | 1,916 | 0.773 |
|  | 3rd | | 2,037 | 0.719 | 1,019 | 4,070 | 0.044 |
| **ASL** | NA (ref) | | | | | | |
|  | AV e BE | | 0.275 | 0.219 | 0.058 | 1,305 | 0.104 |
|  | SA | | 1,006 | 0.544 | 0.348 | 2,903 | 0.990 |
|  | CE | | 1,514 | 0.702 | 0.610 | 3,759 | 0.371 |
|  | Other | | 0.900 | 0.388 | 0.386 | 2,096 | 0.808 |
| **HPV Invitation** | No (ref) | | | | | | |
|  | Yes | | 2,808 | 0.919 | 1,537 | 5,330 | 0.002 |
| **Model II - Sensitivity Analysis** | | | | | | | |
| **Sex** | Male (ref) | |  |  |  |  |  |
|  | Female | | 6,819 | 2.248 | 3,573 | 13,013 | 0.000 |
| **Age** |  | | 0.802 | 0.049 | 0.710 | 0.905 | 0.000 |
| **Smoking habit** | Non-smoker (ref) | | |  |  |  |  |
|  | Smoker | | 0.721 | 0.221 | 0.395 | 1,313 | 0.285 |
|  | Former Smoker | | 0.717 | 0.429 | 0.221 | 2,319 | 0.579 |
| **Year of enrolment** | 1st (ref) | |  |  |  |  |  |
|  | 2nd | | 0.885 | 0.359 | 0.399 | 1,961 | 0.764 |
|  | 3rd | | 1,998 | 0.767 | 0.941 | 4,240 | 0.071 |
| **ASL** | NA (ref) | |  |  |  |  |  |
|  | AV e BE | | 0.258 | 0.215 | 0.050 | 1,323 | 0.104 |
|  | SA | | 1,023 | 0.557 | 0.351 | 2,976 | 0.967 |
|  | CE | | 1,475 | 0.703 | 0.579 | 3,754 | 0.415 |
|  | Other | | .891 | 0.393 | 0.376 | 2,113 | 0.795 |
| **HPV Invitation** | No (ref) | |  |  |  |  |  |
|  | Yes | | 2,575 | 0.866 | 1,332 | 4,978 | 0.005 |
| **Knowledge** |  | | 0.983 | 0.**015** | 0.954 | 1,012 | 0.263 |
| **Attitude** |  | | 2,796 | **1.807** | 1,569 | 4,982 | 0.000 |

**Table 1. Results of the logistic multiple regression analysis**. Main analysis: primary multivariable logistic regression model, adjusted for sex, age, academic year, smoking status, and vaccination invitations. Sensitivity analysis: secondary multivariable logistic regression model, adjusted for for knowledge (23-item score) and attitudes (17-item Likert scale score).
